# Explaining the Association Between Fetal Growth and Childhood ADHD Symptoms: Cross-cohort Replication

**DOI:** 10.1007/s10802-022-00971-9

**Published:** 2022-09-17

**Authors:** Niamh Dooley, Colm Healy, Ross Brannigan, David Cotter, Mary Clarke, Mary Cannon

**Affiliations:** 1 Department of Psychiatry, Royal College of Surgeons in Ireland, Dublin, Ireland; 2 Data Science Centre, Royal College of Surgeons in Ireland, Dublin, Ireland; 3 Department of Psychology, Royal College of Surgeons in Ireland, Dublin, Ireland; 4 Department of Psychiatry, Beaumont Hospital, Dublin, Ireland

**Keywords:** Birth weight, Fetal growth restriction, ADHD, Fetal development, Pregnancy complications

## Abstract

The association between restricted fetal growth and symptoms of attention deficit/hyperactivity disorder (ADHD) in childhood is well-replicated and robust. However, fetal growth is determined by many prenatal factors and associations with mental health may be confounded by familial and social context. In this study, we sought to quantify the relative contributions of prenatal factors and familial confounds to the association between fetal growth and ADHD symptoms. Two independent cohorts were analyzed, the Adolescent Brain Cognitive Development study (ABCD; United States) and the Growing Up in Ireland (GUI) study. ADHD symptoms were measured by the Child Behavior Checklist (ABCD) and the Strengths & Difficulties questionnaire (GUI) at age 9–10. Using sequential regression models, we assessed the change-in-association between fetal growth and ADHD symptoms after controlling for sex, familial factors (socioeconomic/demographic factors & family psychiatric history) and prenatal factors (pregnancy complications & maternal substance-use during pregnancy). Converging findings from cohorts suggested that over a quarter of the association between fetal growth and ADHD symptoms is attributable to familial confounds. The degree to which the association was explained by prenatal factors differed by cohort—pregnancy complications explained a larger proportion of the effect in ABCD (7.9%) than GUI (2.7%), and maternal substance-use explained a larger proportion of the effect in GUI (22.7%) compared to ABCD (4.8%). Different explanations of the fetal growth-ADHD association across cohorts suggests cohort-specific, and potentially nationally-specific, risk factors for fetal growth and related neurodevelopmental outcomes. The evidence suggests early prevention of ADHD in Ireland should focus on minimizing maternal smoking during pregnancy. In the US, prevention and treatment of pregnancy complications are highlighted as viable targets for intervention.

**Supplementary Information:**

The online version contains supplementary material available at 10.1007/s10802-022-00971-9.

## Introduction

Well established risk factors for childhood attention-deficit hyperactivity disorder (ADHD) include genetic/familial susceptibility to ADHD, male sex, and restricted fetal growth (Thapar et al., 2013). As a partially environmentally-determined characteristic, fetal growth may be the most amenable to intervention. However, it is not currently understood which fetal growth determinant best explains the association between restricted fetal growth and ADHD symptoms.

Birth weight is a crude approximation of the quality of fetal growth. If an infant’s weight is appropriate for their gestational age, we could assume that their fetal growth rate was typical. The association between lower birth weight and attention problems in childhood has been replicated many times, in many populations (meta-analyses: Franz et al., 2018; Momany et al., 2018). Lower birth weights, even among those born at full term, are linked with an elevated risk of ADHD and academic difficulties (Class et al., 2014; Groen-Blokhuis et al., 2011; Hultman et al., 2007; Momany et al., 2018). Within-twin and within-sibling studies suggest that the association may be independent from genetics and other familial factors (Class et al., 2014; Ficks et al., 2013; Groen-Blokhuis et al., 2011; Hultman et al., 2007; Lim et al., 2018; Pettersson et al., 2015). Experimentally-induced fetal growth restriction in animals has also been shown to influence neurodevelopment and behavior (Lauritz et al., 2012; Meyer et al., 2014). It has therefore been proposed that poor fetal growth is an independent causal factor in the development of ADHD. The replicable association between birth weight and ADHD symptoms in twins also shows that, despite experiencing the same prenatal exposures (e.g., maternal smoking), twin differences in fetal growth can occur and it is *this* that predicts differences in childhood ADHD symptoms. Large-scale studies of singleton births are needed to understand *which* prenatal factors contribute to restricted fetal growth, and which account for the association between fetal growth and ADHD symptoms on a population level.

The *fetal origins hypothesis* (and more recently the *Developmental Origins of Health and Disease hypothesis*) posits that conditions in utero can permanently program certain aspects of the child’s physiology, explaining why lower birth weights may be causally related to neurodevelopmental issues. However, fetal growth rate and birth weight are the products of many prenatal and pre-pregnancy factors such as maternal malnutrition, smoking, drug-use, certain medications, parental age and pregnancy/gestational complications (Budree et al., 2017; Heaman et al., 2013; McCowan & Horgan, 2009), several of which are also directly associated with subsequent mental health of the child (He et al., 2020; Smith et al., 2016). Proposed mechanisms to explain how prenatal risk factors such as maternal smoking and gestational infection directly impact neurodevelopment have included: lack of oxygen and blood to the fetus leading to altered gene expression (Smith et al., 2016), neurotransmitter function (Laplante et al., 2012), and activation of immune and inflammatory processes (Meyer et al., 2009; Mirza et al., 2015). It may be that birth weight is a convenient proxy for such direct effects of fetal adversity on neurodevelopment. In this study, we quantify the extent to which the association between fetal growth and ADHD symptoms are explained by prenatal determinants of fetal growth. We are particularly interested in the contribution of modifiable prenatal factors such as maternal substance-use in pregnancy. Understanding the extent to which the association is modifiable will help establish the viability of prenatal interventions.

Familial background is a major source of confounding in the theoretically causal pathway between prenatal adversity, lower birth weight and childhood mental health problems. For instance young maternal age is linked with both low birth weights (Aras, 2013) and increased ADHD disruptive behaviours in the child (Tearne, 2015). Household socioeconomics, often exemplified by income and education level, has also shown strong links with both reduced birth weight (Madden, 2014; Martinson & Reichman, 2016) and ADHD risk (Russell et al., 2016). That said, the association between birth weight and ADHD symptoms typically survives correction for familial socioeconomic factors (Abel et al., 2010; Class et al., 2014; Lærum et al., 2017; Pettersson et al., 2019). Low birth weight and ADHD are also not randomly distributed across racial, ethnic or migrant groups. Black women born in the US are more likely to have a low birth weight/preterm child than any other race/ethnicity even after controlling for income and educational attainment, though other unmeasured inequalities cannot be ruled out (Catov et al., 2015; Giscombé & Lobel, 2005). Family psychiatric history is also relevant to both low birth weight and ADHD via several plausible pathways. It captures genetic susceptibility to ADHD (Thapar et al., 2013), but psychiatric issues in the mother may also affect fetal growth via increased stress (Mongan et al., 2019; Wadhwa et al., 2011), and problematic behaviors and coping styles may also be socially learned. Demographic/socioeconomic factors and family psychiatric history should therefore be controlled for when assessing the effect of fetal growth on ADHD symptoms.

No study to our knowledge has assessed the extent to which the association between fetal growth and ADHD symptoms is explained by preceding prenatal factors. This is important information because fetal growth, estimated from weight and age at birth, is not in itself a modifiable factor. Understanding *which* prenatal factors and to what extent they contribute to the association between fetal growth and ADHD symptoms may help identify prenatal targets for preventative interventions. Similarly, but not equivalently, studies have used prenatal factors to directly predict ADHD diagnosis, where the goal is predictive accuracy rather than explanation (Huhdanpaa et al., 2021; Schwenke et al., 2018; Sciberras et al., 2011; Silva et al., 2014; Willoughby et al., 2020). Other research has assessed the effect of birth weight on ADHD symptoms after adjustment for prenatal factors, however the focus of those studies is generally on the final, fully adjusted, birth weight effect, and not on how it changes due to the addition of each type of explanatory factor (Murray et al., 2016; Silva et al., 2014; Wiles et al., 2006). This study attempts to disentangle potentially confounding factors such as familial income and psychiatric history, and prenatal factors, which temporally precede fetal growth and may *explain* its effects.

Several other aspects of our study design differentiate it from the existing literature. First, we use a continuous parent-reported scale as our outcome, rather than binary ADHD diagnosis, or a cut-off corresponding to clinical risk, which have typically been the outcomes of choice when assessing prenatal predictors. While diagnosis can be clinically informative, subjective symptom scales can offer greater sensitivity to smaller effects. This may be particularly important given that low birth weight has been associated with ADHD symptom shifts within the sub-clinical range (Milberger et al., 1997; Murray et al., 2016, 2021). Second, unlike studies which assess one risk factor in isolation (e.g., maternal smoking), our data includes an array of pregnancy complications and substances consumed by the mother, allowing us to assess independence of their effects. Finally, our study uses two independent population-based cohorts of children (both aged 9–10), one from the United States (Adolescent Brain Cognitive Development Study; ABCD; N = 8,358) and one from Ireland (Growing Up in Ireland study; GUI; N = 7,724) to test replicability of findings. Meta-analytic findings suggested the association between birth weight and ADHD symptoms was significantly moderated by geographic region suggesting that context may be important when considering this particular association (Momany et al., 2018). Parallel analysis in two nationally-representative samples may help disentangle generalizable from context-specific explanations for the association between fetal growth and ADHD symptoms.

## Aims of the Study

1. Quantify the extent to which (i) familial confounds and (ii) prenatal factors account for the association between birth weight and childhood ADHD symptoms
2. Assess the cohort-generalizability of these findings.

## Material and Methods

### Participants

#### ABCD (United States)

The Adolescent Brain Cognitive Development (ABCD) Study (abcdstudy.org) is an ongoing longitudinal study of over 10,000 children born 2007–2009 and recruited from 22 sites across the United States. The ABCD sample was designed to mirror US population demographics by recruiting through geographically, demographically, and socioeconomically diverse school systems surrounding each of the research sites. A stratified probability sample of schools was selected for each site based on sex, race/ethnicity, socioeconomic status, and urbanicity to minimize systematic sampling biases in recruitment at the school level (Garavan et al., 2018). We included baseline data only (release 3.0) which assesses the children aged 9–10 (referred to as 9 for simplicity). All data, including details about the pregnancy and birth, was provided retrospectively at this time point. In cases where 2 or more children from the same household were included, we included only the eldest to ensure independence of observations. Our final dataset from ABCD included 8,835 singleton-born children with outcome data (mean age = 119 months [9 years & 11 months], SD = 7 months). The University of California at San Diego (San Diego, CA, 208 USA) Institutional Review Board was responsible for the ethical oversight of the ABCD study. ABCD data can be accessed by application to the NIMH Data Archive (NDA) Repository (ndar.nih.gov). The data used in this report was drawn from Release 3.0 and will be available on the NDA (https://doi.org/10.15154/1524734).

#### GUI (Ireland)

The Growing Up in Ireland (GUI) study (growingup.ie) is an ongoing longitudinal study of over 10,000 children born between December 2007 and June 2008 inclusive. Children were sampled from the Child Benefit Register and stratified by marital status, county of residence, nationality and number of children in household to achieve national representativeness (Quail et al., 2011). Thornton et al. (2013) showed that the proportions of unemployed parents, non-nationals, specific maternal age groups, and parental education levels in the baseline GUI sample approximated those in the Irish population during those years. Data collection began at 9 months of age, with follow-ups at ages 3, 5, 7 and 9 years. We used the 9-month data for information surrounding the pregnancy/birth and socioeconomic factors in the household and the 9-year data to assess ADHD symptoms. For consistency with ABCD, socioeconomic factors such as income and parental education were extracted from the 9-year data. Our final dataset from GUI included 7,724 singleton-born children with outcome data (mean age = 113 months [9 years & 5 months], SD = 2 months). Ethical approval for GUI was granted by the research ethics committee of the Health Research Board in Ireland. Results in this report are based on analyses of data from the Research Microdata Files. This data is not publicly available but is available to researchers affiliated with an Irish institution via application to the Central Statistics Office (see growingup.ie).

For both data sets, the respondent of interviews and questionnaires was most often the mother (ABCD: 85%; GUI: 97%).

#### Cohort Comparison

Cohorts differed in several ways making it inappropriate to merge them. First, ADHD symptoms were measured using two different tools (CBCL & SDQ). Second, information about the pregnancy and birth were recorded 9 months post-partum in GUI and 9 years post-partum in ABCD. Third, some variables had slightly different definitions across cohorts (e.g., family psychiatric history; Table S7).

Ethical approval for the secondary analysis of both datasets was granted by the Research Ethics Committee of the Royal College of Surgeons in Ireland.

## Measures

### ADHD Symptoms

The attention problems subscale of the Child Behavior Checklist (CBCL; Achenbach & Rescorla, 2001) was used in ABCD. This parent-rated questionnaire contains 119 items in total, 10 of which reflect attention problems. Items are rated on a 3-point Likert scale (0 = not at all true; 1 = somewhat true; 2 = very true). The attention problems scale reflects both inattention and hyperactivity symptoms and ranges from 0–20. Meta-analysis shows that this scale can discriminate ADHD cases and controls with moderate accuracy (AUC = 0.82; Chang et al., 2016).

In GUI, the hyperactivity scale from the Strengths and Difficulties Questionnaire was used (SDQ; Goodman, 1997), which is also parent-rated with each item scored on a 3-point Likert scale (0 = not true; 1 = somewhat true; 3 = certainly true). The SDQ contains 25 items, 5 of which form a hyperactivity score. Despite the name, items probe inattention (2), hyperactivity (2) and impulsivity (1). A review of 8 studies showed this scale can discriminate ADHD cases and controls with high accuracy (AUC = 0.90, range = 0.76–0.97; Stone et al., 2010).

The parent-reported CBCL and SDQ attention/hyperactivity problem scales are highly correlated (*r* ~ 0.68; Koskelainen et al., 2000; Stone et al., 2010). Items used to form each scale are provided in Table S1. Raw scores were used rather than T-scores allowing greater precision as they are not truncated, an approach which is advocated by Achenbach and Rescorla (2001) for research in non-clinical settings.

Both scales were log-transformed for analysis due to positive skew.

### Fetal Growth

Fetal growth was calculated as the difference between an individual’s birth weight (in kilograms) and the average birth weight for their gestational age (in weeks). It is therefore a measure of deviance from the average birth weight for a given gestational age. Fetal growth was used instead of raw birth weight as several studies suggest that the fetal growth rate is the aspect of birth weight most strongly linked with ADHD symptoms (Groen-Blokhuis et al., 2011; Momany et al., 2018; Pettersson et al., 2015).

In ABCD, gestational age was not recorded for births after gestational week 40. Therefore, fetal growth does not account for post-term growth in this cohort. Birth weight was originally reported in pounds and ounces in ABCD, which was converted kilograms. Where pounds were reported whilst ounces were not birth weight was calculated to the nearest kg using pounds alone. Values which were unlikely to reflect true birth weight (e.g. ounces only) were removed. Birth information was reported retrospectively at age 9–10 in ABCD, however agreement between medical records and retrospective maternal report of exact birth weight has been shown to be strong (Rice et al., 2007).

## Familial Factors

### Socioeconomics & Demographics

Five socioeconomic & demographic factors were included: race/ethnicity, maternal age, household income, parental education level and single parenthood. Race/ethnicity groups differed by cohort with 5 available in ABCD and 4 available in GUI (Table 1). “Other/mixed” race/ethnicity was not included in GUI analysis due to small group size (n = 29). Maternal age at birth was recorded retrospectively in ABCD while GUI asked the mother’s age at time of first assessment at 9 months postpartum. Household income in ABCD was a choice of 10 income brackets from < $5000 to $200,000 + and reflects income from all sources before deductions. In GUI, income reflected that from all sources, after tax and social insurance, divided by the number of people in the household, and split into deciles. Parental education refers to the education level of the parent with the highest level. Educational levels were binned into 5 levels for both cohorts reflecting (1) incomplete schooling (before completion of secondary/high school); (2) school complete (e.g. Leaving Certificate, high school degree, GED); (3) sub-Bachelor qualification (e.g. non-degree certificate, associate degree); (4) Bachelor or Professional degree; (5) Postgraduate degree, diploma or certificate. In ABCD, dual-parenthood was defined as the presence of a partner to the primary caregiver who was involved in 40% or more of the child’s daily activities. In GUI, dual-parenthood was defined by spouse/partner to the primary caregiver who was resident in the home. Income, education and single-parenthood were recorded at the time of ADHD symptom assessment (age 9).

**Table 1:** Descriptive statistics for all variables used in analysis from both cohorts

**ABCD (United States; N = 8,835)**		**GUI (Ireland; N = 7,724)**	
**Attention-deficit/Hyperactivity Symptoms**			
CBCL “attention problems”	M = 3.15, SD = 3.58 (range: 0–19)	SDQ “hyperactivity difficulties”	M = 3.06, SD = 2.58 (range: 0–10)
Child age (months)	M = 119, SD = 7 (range: 108–131)	Child age (months)	M = 113, SD = 2 (range: 111–120)
**Birth Data**			
Fetal Growth	M = 0.0, SD = 0.5 (range: -2.3—2.5)	Fetal Growth	M = 0.0, SD = 0.5 (range: -2.9—4.3)
Sex	53% male (n = 4,675)	Sex	50% male (n = 3,883)
**Demographics & Socioeconomics**			
Race/Ethnicity of *child*	49% White (n = 4,334)	Race/Ethnicity of *mother*	85% White Irish (n = 6,517)
	23% Hispanic (n = 1,985)		10% White non-Irish (n = 802)
	15% Black (n = 1,348)		2% African or Black (n = 187)
	3% Asian (n = 225)		2% Asian (n = 171)
	11% Other (n = 932)		0.4% Other/Mixed (n = 29)
Maternal age (years)	M = 29, SD = 6 (range: 13–60)	Maternal age (years) ^a^	M = 32, SD = 5 (range: 16–55)
Household income bracket	M = 7.1, SD = 2.5 (range: 1–10)	Household equivalized income deciles	M = 5.8, SD = 2.9 (range: 1–10)
Parent education level	M = 3.5, SD = 1.3 (range:1–5)	Parent education level	M = 3.3, SD = 1.2 (range: 1–5)
Single-parent	20% (n = 1,747)	Single-parent	9% (n = 732)
**Family Psychiatric History** ^b^			
Parental depression/anxiety	34% (n = 3295)	Parental depression/anxiety	34% (n = 2,589)
Parental conduct issues	14% (n = 1314)	Parental trouble with law	15% (n = 1,157)
Familial substance-use issues	20% (n = 1966)	Familial substance-use issues	2% (n = 153)
**Pregnancy Complications**			
Total pregnancy complications	M = 0.5, SD = 0.8 (range: 0–7)	Total pregnancy complications	M = 0.6, SD = 0.9 (range: 0–6)
Severe/persistent nausea and vomiting ^c^	14% (n = 1,163)	Persistent vomiting or nausea	16% (n = 1,279)
Urinary tract infection	8% (n = 651)	Urinary or kidney infection	14% (n = 1,071)
Pre-eclampsia, eclampsia or toxemia	6% (n = 486)	Pre-eclampsia	7% (n = 516)
High blood pressure	9% (n = 727)	High blood pressure	10% (n = 809)
Diabetes (gestational)	7% (n = 575)	Diabetes (gestational)	3% (n = 213)
Heavy bleeding requiring bed rest or special treatment	3% (n = 274)	Bleeding in second half	6% (n = 445)
Rhesus incompatibility	3% (n = 219)	Rhesus incompatibility	4% (n = 300)
Placental previa, abruptio or other issue	3% (n = 228)	Placenta previa or other placental disorder	3% (n = 232)
**Maternal substance-use in pregnancy**			
Smoking	14% (n = 1,244)	Smoking	16% (n = 1,175)
Alcohol	28% (n = 2,293)	Alcohol	22% (n = 1,653)
Drugs ^d^	8% (n = 700)	Drugs ^d^	1% (n = 79)

^a^Maternal age in GUI refers to age at 9 months postpartum

^b^See Table S7 for specific definition of family psychiatric history variables

^c^Specifically defined as nausea and vomiting beyond 6th month or accompanied by weight loss

^d^ABCD drug-use refers to any non-prescription drug; GUI drug-use refers specifically to use of marijuana/cannabis, heroin/methadone/crack/cocaine, or amphetamines/other stimulants

### Family Psychiatric History

Parental depression/anxiety was defined as at least one parent having ever suffered from depression, nerves or nervous breakdown (ABCD), or at least one parent having ever been treated for clinical depression, anxiety, nerves or phobias (GUI). Parental conduct issues in ABCD were captured by either parent having trouble with the police or law, difficulty retaining employment, or getting into fights. In GUI, parental conduct issues were captured by either parent having been in trouble with the police for any offence other than a traffic offence. Familial substance-use problems in ABCD referred to any member of the immediate family (biological parents or full-siblings) ever having a history of drug/alcohol-related problems while in GUI was defined as the study child experiencing alcoholism or drug-use within the immediate family. Specific definitions of each aspect of psychiatric history are provided in Table S7. For GUI, family psychiatric data was aggregated across all available waves to better approximate “lifetime” parental histories (see Supplementary Material for further details).

## Prenatal Factors

### Pregnancy Complications

The eight pregnancy complications recorded in both cohorts were: persistent nausea/vomiting, pre-eclampsia, high blood-pressure, urinary tract (or kidney) infection, bleeding, placenta issues (e.g., previa), rhesus incompatibility and gestational diabetes (insulin-/diet-treated). Table 1 shows any cohort differences in wording of these conditions. Pregnancy complications were retrospectively reported 9–10 years post-partum in ABCD, however long-term maternal recall for obstetric complications has shown to be accurate (Ramos et al., 2020; Rice et al., 2007). As many of these complications may co-occur, a sum of all complications was used for the analysis.

### Maternal Substance-use in Pregnancy

Maternal smoking, alcohol-use and drug-use referred to those events at any point during pregnancy and at any frequency. While frequency and timing of smoking in pregnancy may be relevant (Brannigan et al., 2020) these were not included in the analysis due to cohort-differences in how timing was captured (trimesters 1–3 [GUI] Vs before and after knowledge of the pregnancy [ABCD]; Table S8). In ABCD, drug-use during pregnancy was defined broadly by “any non-prescription drug use”. On the other hand, GUI mothers were asked specifically about use of the following drug groups during pregnancy: cannabis/marijuana, amphetamines or other stimulants, heroin/methadone/crack/cocaine.

These variables may overlap to some degree with family history of substance-use problems (above) however the former is distinct by being specific to the mother during the pregnancy and including any level of substance-use (not necessarily abuse).

## Data Analysis

### Data Cleaning

Coherence between the cohorts was maximized regarding choice of variables, manipulation of variables (e.g., centering, transforming) and statistical analysis. For instance, only those family psychiatric history variables and pregnancy complications that were available in both cohorts were included.

Dimensionality of the data was reduced by creating totals (total number of gestational complications) or overarching binary variables (e.g., *any* drug during pregnancy). This was preferred to data-driven reduction methods such as principal components analysis as it allowed easier comparison between cohorts and generalizability to other studies.

All binary variables were treatment-coded [0,1] except sex which was deviation-coded [-0.5, 0.5] such that the intercept referred to the average of males and females. Continuous predictor variables with non-zero means were centered (income, education, maternal age). Reference levels of categorical variables were the most common level.

### Statistical Analysis

“Explanation” of the association between fetal growth and ADHD symptoms in this study refers to the statistical reduction of the association due to the step-by-step addition of other variables, as with a classic confounding model. A baseline association between fetal growth and ADHD symptoms is established (represented by the beta coefficient) in each cohort while controlling for sex (Model 0; M0). In the next step (M1), 5 potentially confounding socioeconomic and demographic factors are consecutively added to the baseline model and the change in birth weight coefficient from baseline is noted. If the effect of fetal growth reduces in magnitude, there is an overlap or covariance between the contribution of socioeconomic/demographic factors and the contribution of fetal growth to ADHD symptoms. The same logic is applied to family psychiatric history (M2), pregnancy complications (M3) and maternal substance-use in pregnancy (M4). Any remaining effect of fetal growth reflects that which is not explained by variables in M1-4. Order of entry was based on the modifiability of factors with familial factors considered least modifiable and maternal behaviors during pregnancy most modifiable.

Models M0-M4 were standard linear regressions, performed using the *stats* package (v3.6.3) in R. As the outcome (ADHD symptoms) was skewed in both cohorts (Fig. S1), they were log-transformed. As such, B coefficients in these linear models refer to change in log(ADHD symptoms) for each unit increase in predictor, however we were more interested in the % change in estimate due to the addition of other factors (M1-4) rather than the absolute estimate.

To test whether results were robust to a different choice of model, an ordinal logistic model was performed with symptom *groups* as outcome (low, moderate, high or very high; Fig. S1), using the *polr* function from the *MASS* package (v7.3–51.4). Groups were based on percentiles (low =  < 50th percentile, moderate = 50th-79th percentile, high = 80th-89th percentile; very high = 90th-100th percentile). While the coefficients of a logistic model are expressed in log(odds of outcome) the % change in coefficients due to the addition of other factors are comparable to a linear model.

## Results

### Descriptive Statistics

Table 1 provides descriptive statistics for all variables used in the analysis. The average score on ADHD symptom scales was approximately 3 points in both cohorts, despite use of different scales with different ranges (CBCL: M = 3.15 [SD = 3.58]; SDQ: M = 3.06 [SD = 2.58]). Mean fetal growth was zero by definition, with a SD of 0.5 kg in both cohorts. That is, most participants had a birth weight that was typical for their gestational age, but variation by half a kilogram was common. For both cohorts, fetal growth distributions were approximately normal (Fig. S5) and trajectories of fetal growth approximated international norms (Fig. S6).

Racial/ethnic variation was greater in ABCD compared to GUI, the latter of which was 85% White Irish (Table 1). The average maternal age at birth was 2–3 younger in ABCD than in GUI. Mean parental education level was similar across ABCD (M = 3.5, SD = 1.3) and GUI (M = 3.3, SD = 1.2) which was between a non-degree qualification (level 3) and a primary degree (level 4; e.g. Bachelor). Single-parent households were more common in ABCD (20%) compared to GUI (9%). Income levels could not be directly compared across cohorts as ABCD reported income bracket before tax and GUI reported income decile after tax.

### Inferential Statistics

**Fig. 1:**
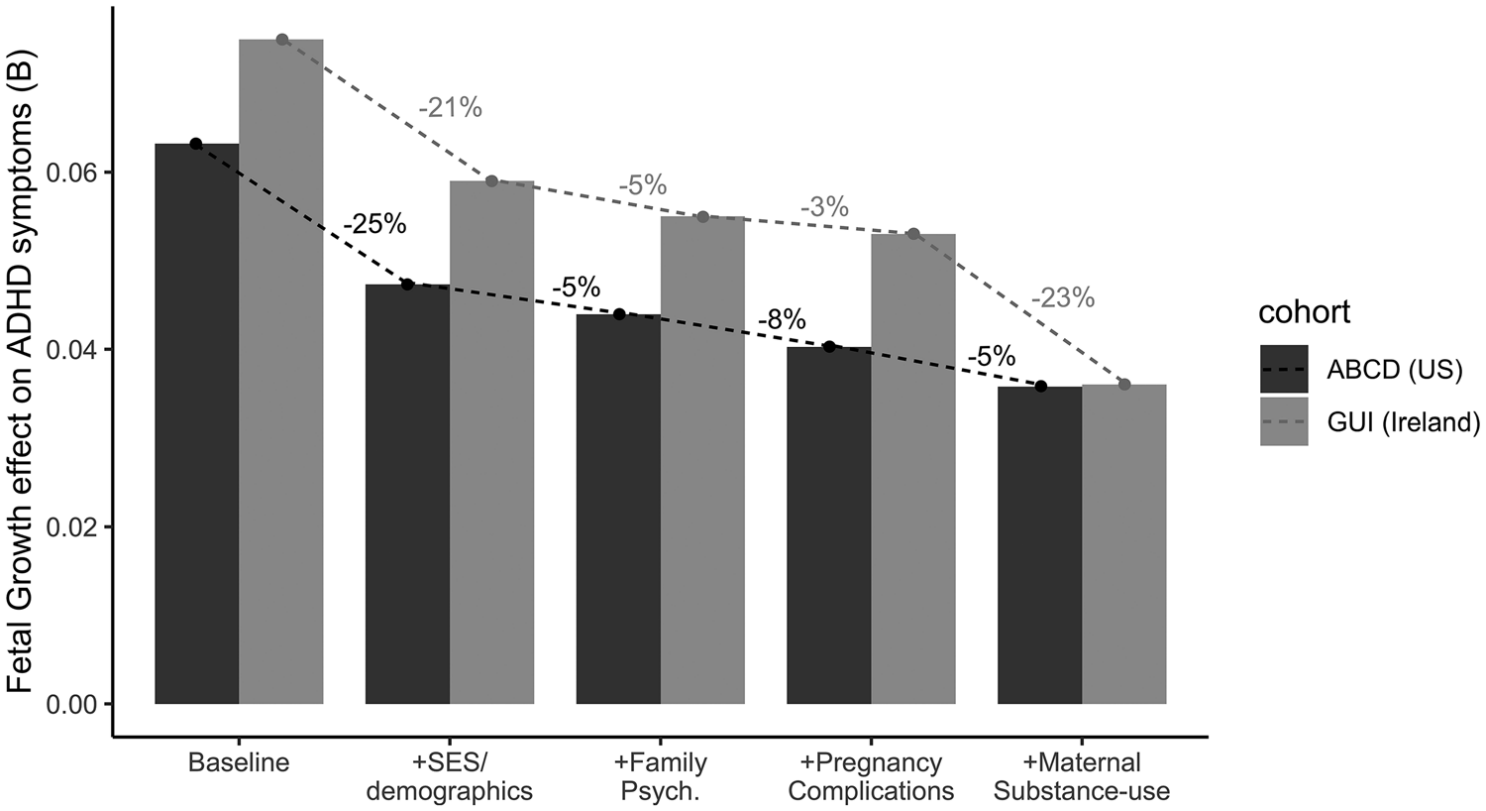
Reduction in the effect of fetal growth on age 9 ADHD symptoms after controlling for socioeconomics/demographics, family psychiatric history, pregnancy complications and maternal substance-use in pregnancy. Hatched trendlines show the gradual decrease in the strength of effect. Percentages reflect the change-in-estimate as a percentage of baseline effect

The baseline association between fetal growth and childhood ADHD symptoms was significant in both ABCD (*B* = 0.06, 95% CI = 0.03–0.10, *t* = 3.41, *p* = 0.001) and GUI (*B* = 0.075, 95% CI = 0.04–0.11, *t* = 4.39, *p* < 0.001). After controlling for socioeconomic and demographic factors (M1), family psychiatric history (M2), pregnancy complications (M3), and maternal substance-use (M4), the effect of fetal growth on ADHD symptoms was reduced to non-significance in ABCD (*B* = 0.04, 95% CI = -0.003 – 0.08, *t* = 1.78, *p* = 0.08) and to marginal significance in GUI (*B* = 0.04, 95% CI = 0.001–0.07, *t* = 1.98, *p* = 0.05). This corresponded to a 43% and 52% drop in the fetal growth effect estimate from baseline to fully-adjusted in ABCD and GUI respectively (Fig. 1). Note B statistics are in terms of log-transformed symptom scales.

**Fig. 2:**
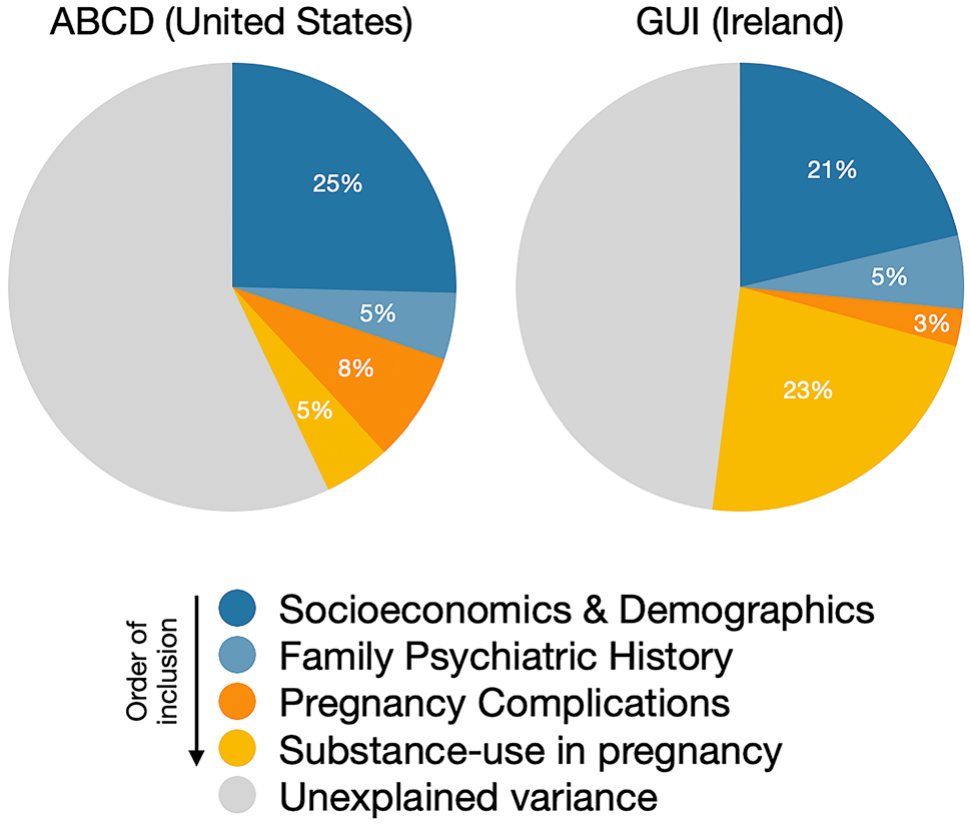
Statistical breakdown of the association between fetal growth and age 9 ADHD symptoms in two cohorts (ABCD and GUI). Segments reflect the proportion of the association attributable to socioeconomics & demographics, family psychiatric history, pregnancy complications and substance-use during pregnancy. Gray portion shows unexplained fetal growth effect on ADHD symptoms

Socioeconomic and demographic factors (household income, parental education, maternal age, single-parenthood, race/ethnicity) accounted for 23.4% of the association between fetal growth and ADHD symptoms on average (ABCD = 25.4% Vs GUI = 21.3%). Family psychiatric history (parent depression/anxiety, parent conduct issues, familial substance-abuse) accounted for a further 5% of the association (ABCD = 4.8% Vs GUI = 5.3%). After controlling for these two sets of confounds (M1 + M2), the association between fetal growth and ADHD symptoms remained significant at the 5% alpha level in both cohorts, though the association was more robust in GUI (*p* = 0.002) compared to ABCD (*p* = 0.027; Table 2). The proportion of the effect explained by M1 + M2 confounds was also higher in ABCD (30.2%) than GUI (26.6%) which is evident from Fig. 2.

**Table 2:** Change in the fetal growth effect on ADHD symptoms, after stepwise control for other factors

		**M0**	**M1**	**M2**	**M3**	**M4**	
		**Baseline model** ^**a**^	**M0 + SES & demographics**	**M1 + Family psych history**	**M2 + Pregnancy complications**	**M3 + Maternal substance-use**	**Total % change**
**ABCD**	**Fetal growth B**	0.063	0.047	0.044	0.039	0.036	
	**95% CI**	[0.03, 0.10]	[0.001, 0.09]	[0.005, 0.08]	[-0.0002, 0.08]	[-0.003, 0.08]	
	***p***	< .001	0.018	0.027	0.051	0.076	
	***η***_***p***_^***2***^	0.001	0.001	0.001	0.001	< 0.001	
	**B change**	—	0.016	0.003	0.005	0.003	
	**% of baseline**	—	25.4%	4.8%	7.9%	4.8%	43%
**GUI**	**Fetal growth B**	0.075	0.059	0.055	0.053	0.036	
	**95% CI**	[0.04, 0.11]	[0.02, 0.09]	[0.02, 0.09]	[0.02, 0.09]	[0.0003, 0.07]	
	***p***	< .001	0.001	0.002	0.003	0.048	
	***η***_***p***_^***2***^	0.003	0.002	0.001	0.001	0.001	
	**B change**	—	0.016	0.004	0.002	0.017	
	**% of baseline**	—	21.3%	5.3%	2.7%	22.7%	52%
**Cohort Ave**	**% of baseline**		**23.4%**	**5.0%**	**5.3%**	**13.7%**	**47%**

*SES* socioeconomic status

ADHD symptom scales were log-transformed

^a^ Baseline model: fetal growth + sex

The next two steps of prenatal factors (pregnancy complications; maternal substance-use) explained an additional 12.7% of the fetal growth effect in ABCD but an additional 25.4% of the effect in GUI. This cohort-difference was predominantly driven by the higher proportion accounted for by maternal substance use in GUI (22.7%) compared to ABCD (4.8%; Table 2; Fig. 2).

The baseline model, including just fetal growth and sex accounted for ~ 3% of the variance in ADHD symptom scores (ABCD *R*^*2*^ = 2.9%; GUI *R*^*2*^ = 3.7%) while the fully-adjusted model (M4) accounted for 8–10% (ABCD *R*^*2*^ = 9.6%; GUI *R*^*2*^ = 8.3%). In ABCD, the most reliable independent predictors of ADHD symptoms were male sex (*t* = 15.19, *η*_*p*_^*2*^ = 0.03), parental depression/anxiety (*t* = 10.84, *η*_*p*_^*2*^ = 0.02) and the number of pregnancy complications experienced (*t* = 7.01, *η*_*p*_^*2*^ = 0.01; Table S3). The most reliable independent predictors of ADHD symptoms in GUI were male sex (*t* = 15.58, *η*_*p*_^*2*^ = 0.04), parental depression/anxiety (*t* = 7.45, *η*_*p*_^*2*^ = 0.01) and younger maternal age (*t* = -7.54, *η*_*p*_^*2*^ = 0.01; Table S5).

An ordinal logistic regression predicting groups of varying ADHD symptom severity (low, moderate, high, very high) was performed to test statistical robustness of linear models. Results were similar regardless of whether linear or ordinal models were used (Table S2; Fig. S4)— changes to the fetal growth effect estimate due to each step of control variables were similar (± 2%) to the linear model with one exception. In ABCD (US), the total proportion accounted for was greater when predicting symptom groups (ordinal 50% vs linear 43%). This was driven by the greater change-in-estimate due to control of socioeconomics/demographic factors in the ordinal model (31.3%) compared to the linear model (25.4%; Table S2).

## Discussion

Results from two independent cohorts suggest the association between fetal growth and ADHD symptoms at age 9 can be explained in part by sociodemographic confounds and prenatal events. Across both cohorts, over 25% of the association was attributable to familial confounds (socioeconomics, demographics, family psychiatric history) and the significance of the association survived adjustment for familial confounds but not prenatal factors. However, results differed by cohort regarding the relative contributions of socioeconomic/demographic factors (ABCD > GUI) and prenatal factors (GUI > ABCD) to the effect of fetal growth on ADHD symptoms (Fig. 2; Fig. S4). Fetal growth, as estimated from weight and age at birth, was a relatively context-independent predictor of ADHD symptoms while the explanatory or driving factors of the association were somewhat context-dependent.

Averaging cohort results (Table 2), 23.4% of the association between fetal growth and ADHD symptoms was captured by socioeconomic and demographic factors such as household income, race/ethnicity, parental age, education and single parenthood. This is consistent with other studies which have found the fetal growth (or small-for-gestational-age) effect on ADHD outcomes attenuates after controlling for factors such as maternal age, household income, parental education or single-parenthood (Murray et al., 2016; Pettersson et al., 2019; Wiles et al., 2006). The decrease in fetal growth effect after control of socioeconomic and demographic factors was slightly greater in ABCD (25.4%) compared to GUI (21.3%), however this cohort-discrepancy widened in an ordinal sensitivity analysis (ABCD 31.3% Vs GUI 20.5%; Table S2). This disparity may be explained by national differences in the correlation between socioeconomic factors and fetal growth. Martinson and colleagues (2016) found that income-related inequalities in low birth weight rates were present in the US, the United Kingdom, Canada and Australia, but that the magnitude of this gradient was greatest in the US. The authors suggested that the more generous social support and healthcare systems of the UK, Canada and Australia may play a buffering role. Differences may also be explained by the greater racial and ethnic heterogeneity in ABCD (Table 1) and the greater correlation between certain race/ethnicities and fetal growth in ABCD compared to GUI (Figs. S2-S3).

Pregnancy complications accounted for 5.3% of the fetal growth effect on ADHD symptoms on average, with a larger proportion accounted for in ABCD (7.9%) than GUI (2.7%). Ordinal models approximated this cohort difference (9.9% vs 3.2%). The mean number of pregnancy complications in mothers across cohorts was similar across cohorts (Table 1) however the independent effect of pregnancy complications on ADHD symptoms was stronger in ABCD compared to GUI (Tables S3, S5). This suggests that, despite being similarly prevalent across cohorts, pregnancy complications have more adverse effects on both fetal growth and childhood behavior in ABCD compared to GUI. While we did not assess interactive effects between pregnancy complications and maternal age, the younger age of ABCD mothers compared to those in GUI may explain the difference in results (Table 1). For instance, the association between preeclampsia and small-for-gestational age is stronger for mothers under 25 compared to over 25 (Li et al., 2018). Alternatively, this result may be explained by unmeasured cohort differences such as in maternal weight status, stress access to prenatal care, or quality of care (Bronstein et al., 2018; Fuchs et al., 2022). Rates of obesity, for instance, are higher among women in the US compared to women in comparable countries, and this may interact multiplicatively with certain pregnancy complications to increase the risk of both restricted fetal growth and neurodevelopmental issues (Bronstein et al., 2018; Kong et al., 2020).

Perhaps the most striking aspect of Fig. 2 is the greater proportion of the effect accounted for by maternal substance-use in GUI (22.7%) compared to ABCD (4.8%). This is despite similar reported rates of smoking, and lower rates of alcohol and drug-use among GUI mothers (Table 1). GUI mothers smoked more cigarettes and more persistently than ABCD mothers, smoking ~ 9 cigarettes per day on average throughout pregnancy (Table S8). Maternal smoking was also a significant *independent* predictor of ADHD symptoms in the GUI, but not ABCD. By contrast, maternal alcohol-use and drug-use during pregnancy were significant independent predictors of symptoms in ABCD, but not in GUI (Tables S3, S5). Our findings are consistent with another analysis of the GUI data which showed strong links between maternal smoking and intrauterine growth restriction in Ireland (Madden, 2014), and support the need for improved smoking cessation programs in Irish maternal hospitals (Reynolds et al., 2017). There has been some evidence that the association between smoking in pregnancy and offspring ADHD is not causal and is fully accounted for by shared genetic factors between mother and child (Rice et al., 2018). Such logic could also be applied to other types of substance-use in pregnancy. However, there *is* evidence supporting the causal association between maternal smoking in pregnancy and birth weight (Rice et al., 2018). Given reliable associations between fetal growth and ADHD symptoms have been observed in human and animal studies (see introduction), it may be that maternal substance-use in pregnancy, such as smoking, impacts child neurodevelopment via fetal growth restriction (Brannigan et al., 2020).

Our results suggest such prenatal factors can capture up to a quarter of the birth weight effect on ADHD symptoms; however future studies will need to assess, in practice, whether reduction in prenatal risks have any tangible effect on childhood ADHD symptoms at a population level. Effect sizes were small– Table 2 indicated that fetal growth accounted for less than 0.5% of variance in ADHD symptoms across all models (all *η*_*p*_^*2*^ < 0.005) and supplementary tables showed that fully-adjusted models explained just 8–10% of variance in outcomes (ABCD R^2^ = 9.6%; GUI R^2^ = 8.3%; Tables S3, S5). A meta-analysis suggested birth weight accounted for 2.25% in the variance in ADHD symptoms (r = -0.15; Momany et al., 2018). While the effect size observed in this study was smaller, this does not necessarily invalidate the clinical relevance of our findings. First, even well-established risk factors for ADHD such as male sex and parental psychopathology had small effect sizes (*η*_*p*_^*2*^ < 0.05). This could be explained by our large sample sizes and inclusion of normative symptom scales rather than groups from each end of the spectrum (Kühberger et al., 2014). Second, we know from animal experimentation (Lauritz et al., 2012; Meyer et al., 2014), human meta-analysis (Momany et al., 2018) and multiple twin studies (Ficks et al., 2013; Groen-Blokhuis et al., 2011; Hultman et al., 2007; Pettersson et al., 2015) that fetal growth is a highly replicable predictor of neurodevelopmental problems, even when genetic and social confounds are controlled for. Third, other large cohort studies have showed birth weight accounts for < 1% of the variance in ADHD symptom dimensions (Ficks et al., 2013; van Mil et al., 2015). Finally, the importance of studying prenatal risks to neurodevelopment lies in their temporal precedence over all postnatal risk factors. Small deviations from typical neurodevelopment (reflected by small effect sizes) at an early stage of development have the potential to moderate the effects of all subsequent insults.

The primary strength of this study is its use of two large nationally-representative cohorts. The matched analysis on both cohorts helps determine which findings are generalizable and which may be cohort- or nation-specific. While Ireland and the US are both developed countries, they differ on demographic make-up, healthcare systems, policies and culture. As some of these largely unmeasured factors may confound the association between fetal growth restriction and childhood psychopathology, replicating the association across cohorts can be considered further support for a causal association (Murray et al., 2016). Other strengths include: thorough control of potential confounds, the use of alternative statistical modelling to probe the robustness of findings (ordinal regression), and the specificity of our target population to children aged 9–10 born between 2007 and 2009.

Several aspects of the study design limit interpretation of results. First, comparability of cohort results is limited by differences in outcome scales (CBCL vs SDQ), in definitions of sociodemographic and family psychiatric variables (see Supplementary Material) and in the gestational age range captured (capped at 40 weeks in ABCD). Future studies assessing the generalizability of prenatal contributions to fetal growth and ADHD symptoms should use samples with better matched data, merged into one analysis, to quantitively assess the significance of cohort differences. Second, in ABCD we rely on the retrospective report of gestational and birth events which may be influenced by recall bias (9 years on). In both cohorts, we rely on the mother to report to provide both exposure and outcome data, which may be biased. Third, the study was conducted in singleton-born children thus results are not applicable to twins. Finally, there may be unmeasured sources of confounding such as migrant status, neighborhood poverty, maternal health, weight and stress levels.

## Conclusions

We found replicable evidence across Irish and US-based cohorts for the linear association between reduced fetal growth and increased ADHD symptoms by age 9. Background familial confounds (e.g. income, race/ethnicity, family psychiatric history) accounted for over a quarter of that association in both cohorts, however it remained statistically significant. Pregnancy complications and maternal substance-use accounted for different proportions of the fetal growth effect across cohorts, which may reflect societal and cultural differences.

## Supplementary Information

Below is the link to the electronic supplementary material.

Supplementary file1 (DOCX 2959 KB)

## Data Availability

ABCD data is stored in the NIMH Data Archive (NDA) Repository (ndar.nih.gov) and can be accessed by application to the NDA. The data used in this report was drawn from Release 3.0 and will be available on the NDA (https://doi.org/10.15154/1524734). GUI data is managed by Irish Department of Children, Equality, Disability, Integration & Youth in association with the Central Statistics Office (CSO). Results in this report are based on analyses of data from the Research Microdata Files. This data is not publicly available but is available to researchers affiliated with an Irish institution via application to the CSO (see growingup.ie).
