# Supplementary material for: Explaining the Association Between Fetal Growth and Childhood ADHD Symptoms: Cross-cohort Replication
Source: Res Child Adolesc Psychopathol. 2022 Sep 17;51(2):247–59. doi: 10.1007/s10802-022-00971-9 (PMC9867674; doi:10.1007/s10802-022-00971-9)
Supplement: Supplementary file 1 — Supplementary file1 (DOCX 2959 KB) [file 10802_2022_971_MOESM1_ESM.docx]

# Supplementary Material

**Figure S1.** Distribution of the ADHD outcome scales in ABCD (CBCL) and GUI (SDQ) colored by group (low= below 50th percentile; moderate= 50th-79th percentile; high=80th-89th percentile; very high= 90th percentile +).
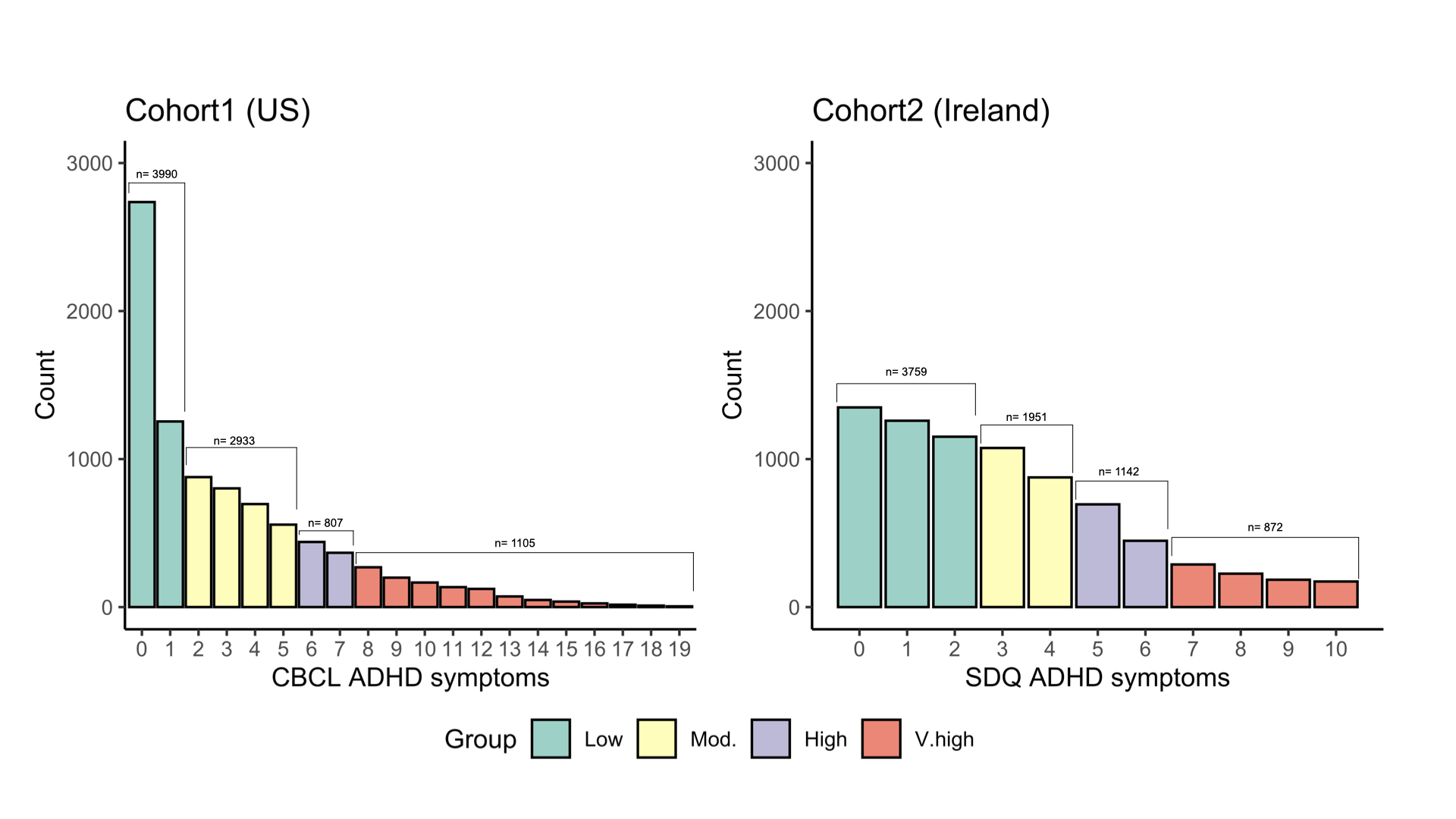


**Table S1.** Constituent items in each outcome scale.

| **ABCD (CBCL)** “…describes your child now or within the past 6 months…” | **GUI (SDQ)** “…on the basis of the child’s behavior over the last 6 months or this school year” |
| --- | --- |
| 1. Acts too young for his/her age | 1. Restless, overactive, cannot stay still for long |
| 2. Fails to finish things he/she started | 2. Constantly fidgeting or squirming |
| 3. Can’t concentrate, can’t pay attention for long | 3. Easily distracted, concentration wanders |
| 4. Can't sit still, restless or hyperactive | 4. Thinks things out before acting (R) |
| 5. Confused or seems to be in a fog | 5. Sees tasks through to the end, good attention span (R) |
| 6. Daydreams or gets lost in his/her thoughts |  |
| 7. Impulsive or acts without thinking |  |
| 8. Poor school work |  |
| 9. Inattentive or easily distracted |  |
| 10. Stares blankly |  |
| R = Reverse coded | |


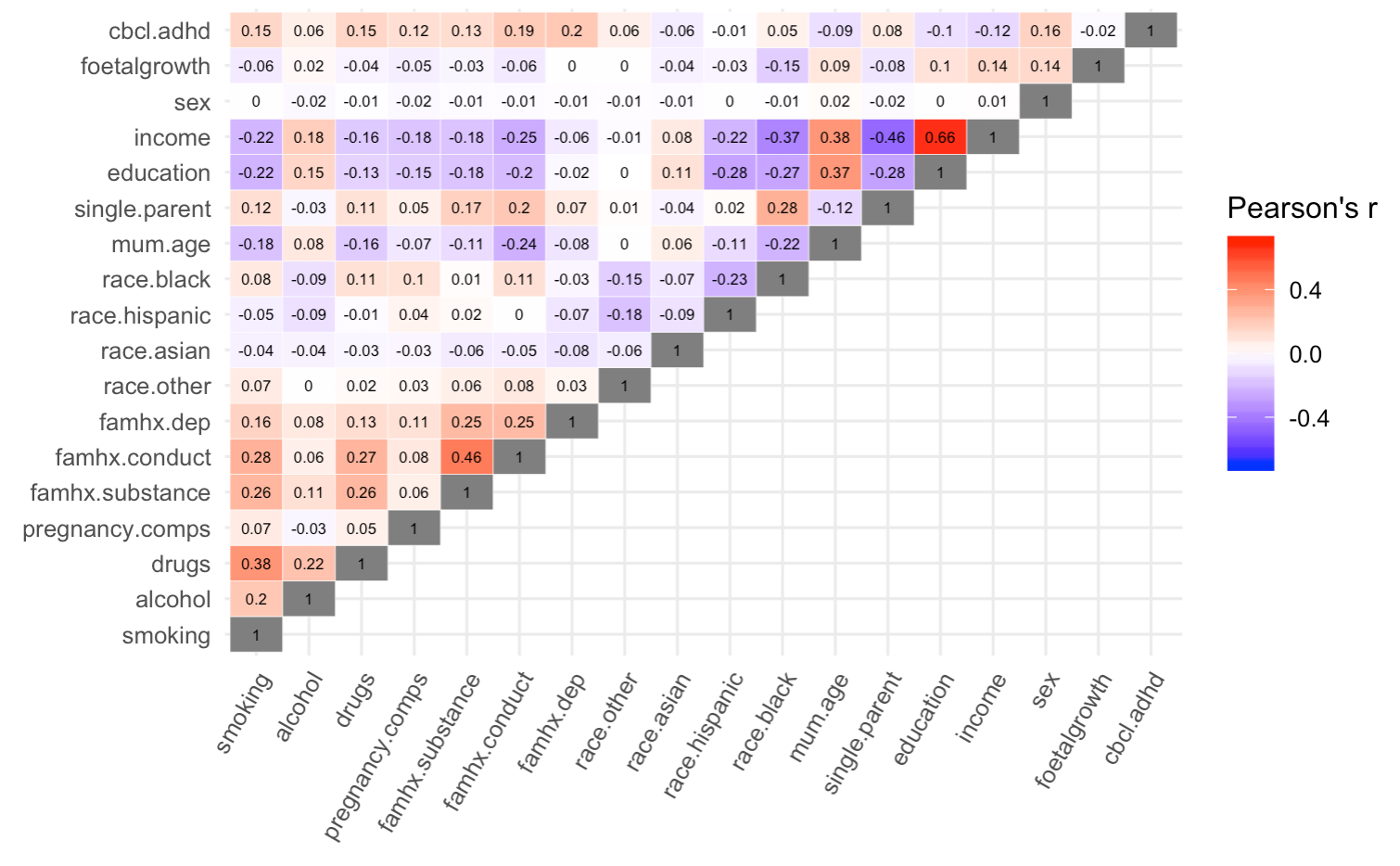
**Figure S2.** Pearson’s correlation heat-map for variables included in ABCD (US) analysis

**Figure S3.** Pearson’s correlation heat-map for variables included in GUI (Ireland) analysis.

pregnancy.comps


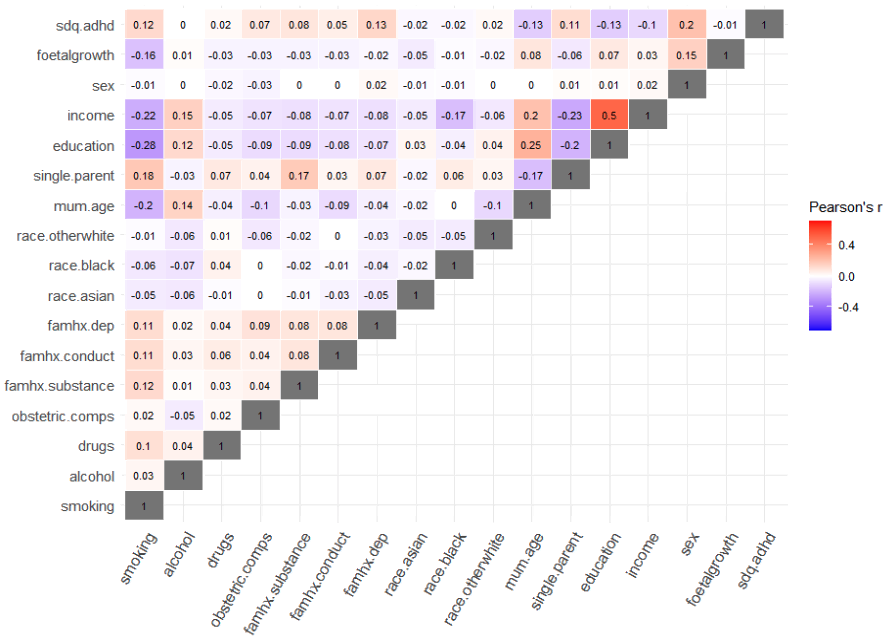

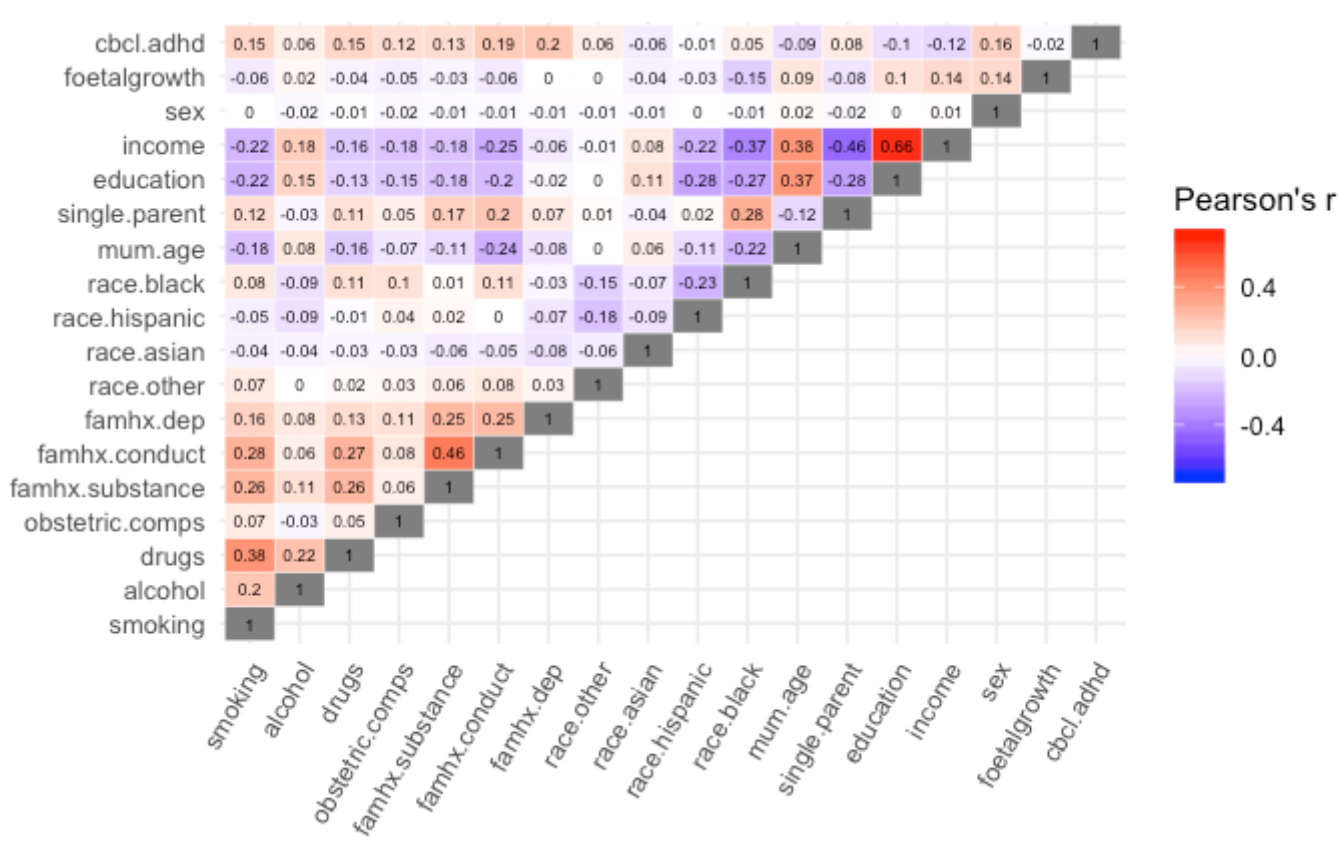


**Table S2.** Change in the fetal growth effect on ADHD symptoms due to sequential control for other factors (M1-4) when outcome is represented as linear (top) Vs as ordinal groups (bottom).

|  |  | **M0*** | **M1** | **M2** | **M3** | **M4** | **Total % change** |
| --- | --- | --- | --- | --- | --- | --- | --- |
|  |  |  | + SES & demographics | + Family psychiatric history | + Pregnancy complications | + Maternal substance-use |  |
| ***Linear Outcome*** *(ADHD scale; log transformed)* | | | | | | | |
| **ABCD (US)** | Fetal growth effect (B, p) | 0.063 | 0.047 | 0.044 | 0.039 | 0.036 |  |
|  |  | *<.001* | *.02* | *.03* | *.05* | *.08* |  |
|  | B change | — | 0.016 | 0.003 | 0.005 | 0.003 |  |
|  | % of baseline | — | 25.4% | 4.8% | 7.9% | 4.8% | 43% |
| **GUI (Ireland)** | Fetal growth effect (B, p) | 0.075 | 0.059 | 0.055 | 0.053 | 0.036 |  |
|  |  | *<.001* | *.001* | *.002* | *.003* | *.05* |  |
|  | B change | — | 0.016 | 0.004 | 0.002 | 0.017 |  |
|  | % of baseline | — | 21.3% | 5.3% | 2.7% | 22.7% | 52% |
| **Cohort Average** | % of baseline |  | **23.4%** | **5.0%** | **5.3%** | **13.7%** | **47%** |
| ***Ordinal group outcome*** *(low, moderate, high or very high symptoms)* | | | | | | | |
| **ABCD (US)** | Fetal growth effect (B, p) | 0.131 | 0.090 | 0.085 | 0.072 | 0.065 |  |
|  |  | *.002* | *.04* | *.06* | *.11* | *.16* |  |
|  | B change | — | 0.041 | 0.005 | 0.013 | 0.007 |  |
|  | % of baseline | — | 31.3% | 3.8% | 9.9% | 5.3% | 50% |
| **GUI (Ireland)** | Fetal growth effect (B, p) | 0.185 | 0.147 | 0.135 | 0.129 | 0.091 |  |
|  |  | *<.001* | *.005* | *.01* | *.01* | *.12* |  |
|  | B change | — | 0.038 | 0.012 | 0.006 | 0.038 |  |
|  | % of baseline | — | 20.5% | 6.5% | 3.2% | 20.5% | 51% |
| **Cohort Average** | % of baseline |  | **25.9%** | **5.2%** | **6.6%** | **12.9%** | **51%** |
| *Baseline model M0: fetal growth + sex  SES: socioeconomic status | | | | | | |  |

**Figure S4.** Breakdown of association between fetal growth and odds of higher group membership (low, moderate, high and very high symptoms). Grey portion shows unexplained fetal growth effect.


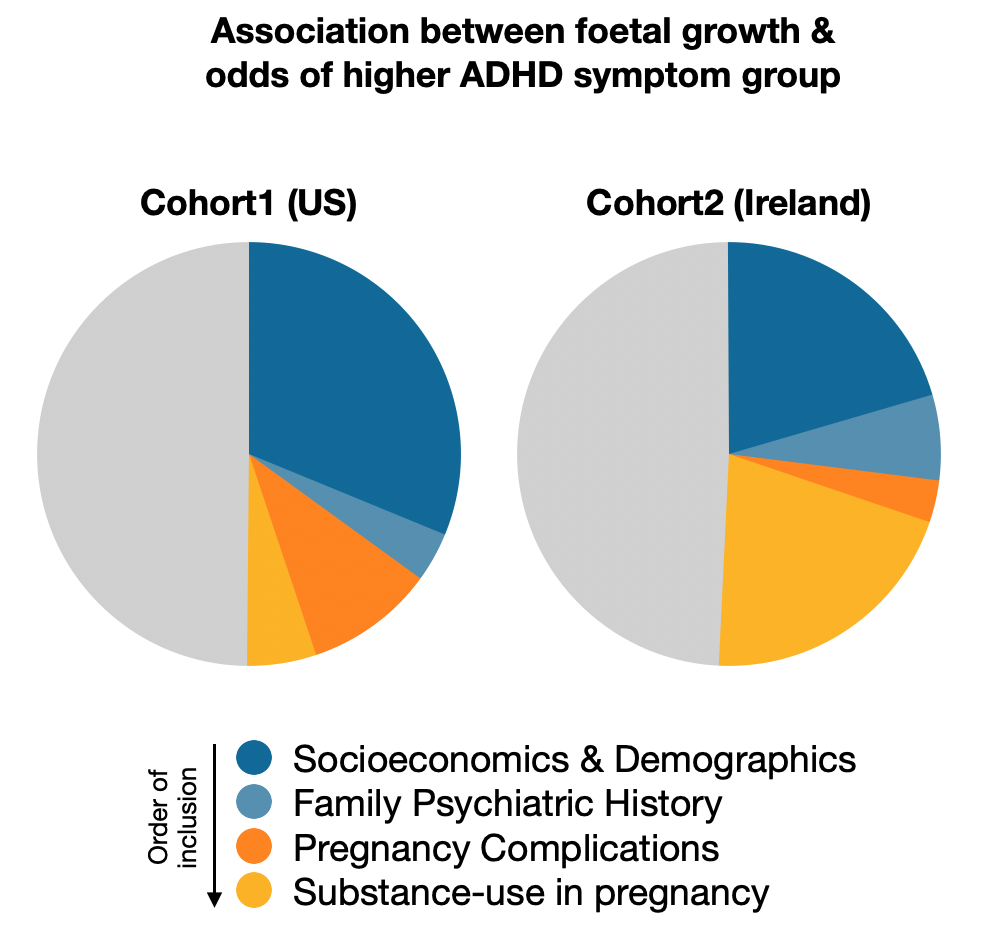


**Table S3.** Results of linear modelling in ABCD (US)

|  | ***Dependent variable: Log-transformed ADHD symptoms (CBCL)*** | | | | |
| --- | --- | --- | --- | --- | --- |
|  | **baseline** | **+SES/Demo** | **+famhx** | **+complications** | **+substances** |
| **Restricted Fetal Growth** | 0.063 (0.019) | 0.047 (0.020) | 0.044 (0.020) | 0.039 (0.020) | 0.036 (0.020) |
|  | t = 3.409 | t = 2.376 | t = 2.219 | t = 1.952 | t = 1.775 |
|  | p = 0.0007 | p = 0.018 | p = 0.027 | p = 0.051 | p = 0.076 |
| **Male sex** | 0.297 (0.019) | 0.295 (0.020) | 0.297 (0.020) | 0.300 (0.020) | 0.302 (0.020) |
|  | t = 15.702 | t = 14.946 | t = 15.201 | t = 15.347 | t = 15.194 |
|  | p = 0.000 | p = 0.000 | p = 0.000 | p = 0.000 | p = 0.000 |
| **Asian** |  | -0.263 (0.068) | -0.174 (0.068) | -0.174 (0.068) | -0.154 (0.068) |
|  |  | t = -3.896 | t = -2.556 | t = -2.561 | t = -2.263 |
|  |  | p = 0.0001 | p = 0.011 | p = 0.011 | p = 0.024 |
| **Black** |  | -0.016 (0.034) | 0.025 (0.035) | 0.010 (0.035) | 0.008 (0.036) |
|  |  | t = -0.468 | t = 0.712 | t = 0.280 | t = 0.217 |
|  |  | p = 0.640 | p = 0.477 | p = 0.780 | p = 0.829 |
| **Hispanic** |  | -0.057 (0.027) | -0.008 (0.027) | -0.013 (0.027) | 0.003 (0.028) |
|  |  | t = -2.099 | t = -0.299 | t = -0.482 | t = 0.097 |
|  |  | p = 0.036 | p = 0.765 | p = 0.630 | p = 0.924 |
| **Other race/ethnicity** |  | 0.113 (0.033) | 0.107 (0.033) | 0.097 (0.033) | 0.106 (0.034) |
|  |  | t = 3.397 | t = 3.199 | t = 2.927 | t = 3.096 |
|  |  | p = 0.001 | p = 0.002 | p = 0.004 | p = 0.002 |
| **Income** |  | -0.024 (0.006) | -0.018 (0.006) | -0.015 (0.006) | -0.017 (0.006) |
|  |  | t = -4.026 | t = -2.918 | t = -2.497 | t = -2.765 |
|  |  | p = 0.0001 | p = 0.004 | p = 0.013 | p = 0.006 |
| **Education** |  | -0.014 (0.011) | -0.010 (0.011) | -0.006 (0.011) | -0.005 (0.011) |
|  |  | t = -1.285 | t = -0.980 | t = -0.601 | t = -0.487 |
|  |  | p = 0.199 | p = 0.327 | p = 0.549 | p = 0.627 |
| **Single-parent** |  | 0.060 (0.028) | 0.006 (0.029) | 0.010 (0.029) | 0.001 (0.029) |
|  |  | t = 2.111 | t = 0.211 | t = 0.353 | t = 0.046 |
|  |  | p = 0.035 | p = 0.833 | p = 0.724 | p = 0.964 |
| **Maternal age** |  | -0.007(0.002) | -0.004 (0.002) | -0.004(0.002) | -0.003 (0.002) |
|  |  | t = -4.018 | t = -2.119 | t = -2.171 | t = -1.596 |
|  |  | p = 0.0001 | p = 0.035 | p = 0.030 | p = 0.111 |
| **Parental depression/anxiety** |  |  | 0.264 (0.021) | 0.248 (0.021) | 0.237 (0.022) |
|  |  |  | t = 12.354 | t = 11.614 | t = 10.844 |
|  |  |  | p = 0.000 | p = 0.000 | p = 0.000 |
| **Parental conduct problems** |  |  | 0.206 (0.034) | 0.197 (0.034) | 0.159 (0.035) |
|  |  |  | t = 6.032 | t = 5.755 | t = 4.512 |
|  |  |  | p = 0.000 | p = 0.000 | p = 0.00001 |
| **Familial substance-use** |  |  | 0.037 (0.028) | 0.036 (0.028) | -0.001 (0.029) |
|  |  |  | t = 1.327 | t = 1.268 | t = -0.044 |
|  |  |  | p = 0.185 | p = 0.205 | p = 0.966 |
| **Total pregnancy Complications** |  |  |  | 0.086 (0.012) | 0.086 (0.012) |
|  |  |  |  | t = 7.106 | t = 7.014 |
|  |  |  |  | p = 0.000 | p = 0.000 |
| **Smoking in pregnancy** |  |  |  |  | 0.061 (0.033) |
|  |  |  |  |  | t = 1.836 |
|  |  |  |  |  | p = 0.067 |
| **Alcohol in pregnancy** |  |  |  |  | 0.098 (0.023) |
|  |  |  |  |  | t = 4.239 |
|  |  |  |  |  | p = 0.00003 |
| **Drugs in pregnancy** |  |  |  |  | 0.197 (0.042) |
|  |  |  |  |  | t = 4.704 |
|  |  |  |  |  | p = 0.00001 |
| **Observations** | 8358 | 7486 | 7310 | 7293 | 6974 |
| **R^2^** | 0.029 | 0.048 | 0.082 | 0.087 | 0.096 |
| **Adjusted R^2^** | 0.029 | 0.047 | 0.080 | 0.086 | 0.094 |

**Table S4.** Results of ordinal logistic modelling in ABCD (US)

|  | ***Dependent variable: ADHD symptom group (low, moderate, high, very high)*** | | | | |
| --- | --- | --- | --- | --- | --- |
|  | **baseline** | **+SES+Demo** | **+famhx** | **+complications** | **+substances** |
| **Restricted Fetal Growth** | 0.131(0.040) | 0.090(0.044) | 0.085(0.045) | 0.072(0.045) | 0.065(0.046) |
|  | t = 3.228 | t = 2.037 | t = 1.895 | t = 1.593 | t = 1.392 |
|  | p = 0.002 | p = 0.042 | p = 0.059 | p = 0.112 | p = 0.164 |
| **Male sex** | 0.621(0.042) | 0.629(0.044) | 0.648(0.045) | 0.655(0.045) | 0.668(0.047) |
|  | t = 14.854 | t = 14.162 | t = 14.315 | t = 14.421 | t = 14.344 |
|  | p = 0.000 | p = 0.000 | p = 0.000 | p = 0.000 | p = 0.000 |
| **Asian** |  | -0.499(0.157) | -0.290(0.163) | -0.297(0.163) | -0.259(0.164) |
|  |  | t = -3.176 | t = -1.781 | t = -1.825 | t = -1.580 |
|  |  | p = 0.002 | p = 0.075 | p = 0.068 | p = 0.115 |
| **Black** |  | -0.023(0.077) | 0.069(0.080) | 0.038(0.080) | 0.041(0.082) |
|  |  | t = -0.293 | t = 0.870 | t = 0.477 | t = 0.501 |
|  |  | p = 0.770 | p = 0.385 | p = 0.634 | p = 0.617 |
| **Hispanic** |  | -0.129(0.061) | -0.007(0.063) | -0.021(0.063) | 0.016(0.065) |
|  |  | t = -2.097 | t = -0.104 | t = -0.333 | t = 0.241 |
|  |  | p = 0.037 | p = 0.918 | p = 0.739 | p = 0.810 |
| **Other race/ethnicity** |  | 0.275(0.074) | 0.267(0.075) | 0.250(0.076) | 0.266(0.079) |
|  |  | t = 3.733 | t = 3.541 | t = 3.295 | t = 3.381 |
|  |  | p = 0.0002 | p = 0.0004 | p = 0.001 | p = 0.001 |
| **Income** |  | -0.058(0.013) | -0.041(0.014) | -0.037(0.014) | -0.040(0.014) |
|  |  | t = -4.335 | t = -3.035 | t = -2.680 | t = -2.849 |
|  |  | p = 0.00002 | p = 0.003 | p = 0.008 | p = 0.005 |
| **Education** |  | -0.030(0.024) | -0.025(0.024) | -0.015(0.025) | -0.010(0.025) |
|  |  | t = -1.239 | t = -1.023 | t = -0.631 | t = -0.396 |
|  |  | p = 0.216 | p = 0.307 | p = 0.529 | p = 0.693 |
| **Single-parent** |  | 0.082(0.063) | -0.029(0.065) | -0.027(0.065) | -0.049(0.067) |
|  |  | t = 1.310 | t = -0.454 | t = -0.421 | t = -0.731 |
|  |  | p = 0.191 | p = 0.650 | p = 0.674 | p = 0.465 |
| **Maternal age** |  | -0.015(0.004) | -0.009(0.004) | -0.009(0.004) | -0.006(0.004) |
|  |  | t = -3.916 | t = -2.161 | t = -2.201 | t = -1.475 |
|  |  | p = 0.0001 | p = 0.031 | p = 0.028 | p = 0.141 |
| **Parental depression/anxiety** |  |  | 0.595(0.049) | 0.566 (0.049) | 0.536 (0.050) |
|  |  |  | t = 12.263 | t = 11.583 | t = 10.674 |
|  |  |  | p = 0.000 | p = 0.000 | p = 0.000 |
| **Parental conduct problems** |  |  | 0.470 (0.076) | 0.455(0.077) | 0.375(0.080) |
|  |  |  | t = 6.145 | t = 5.916 | t = 4.681 |
|  |  |  | p = 0.000 | p = 0.000 | p = 0.00001 |
| **Familial substance-use** |  |  | 0.063(0.064) | 0.057(0.064) | -0.027(0.067) |
|  |  |  | t = 0.992 | t = 0.893 | t = -0.411 |
|  |  |  | p = 0.322 | p = 0.373 | p = 0.681 |
| **Total pregnancy Complications** |  |  |  | 0.188(0.028) | 0.190(0.028) |
|  |  |  |  | t = 6.808 | t = 6.710 |
|  |  |  |  | p = 0.000 | p = 0.000 |
| **Smoking in pregnancy** |  |  |  |  | 0.179(0.075) |
|  |  |  |  |  | t = 2.382 |
|  |  |  |  |  | p = 0.018 |
| **Alcohol in pregnancy** |  |  |  |  | 0.194(0.054) |
|  |  |  |  |  | t = 3.614 |
|  |  |  |  |  | p = 0.0004 |
| **Drugs in pregnancy** |  |  |  |  | 0.446(0.095) |
|  |  |  |  |  | t = 4.690 |
|  |  |  |  |  | p = 0.00001 |
| **Observations** | 8358 | 7486 | 7310 | 7293 | 6974 |

**Table S5.** Results of linear modelling in GUI (Ireland)

|  | ***Dependent variable: Log-transformed ADHD symptoms (SDQ)*** | | | | |
| --- | --- | --- | --- | --- | --- |
|  | **baseline** | **+SES/Demo** | **+famhx** | **+complications** | **+substances** |
| **Restricted Fetal Growth** | 0.075 (0.017) | 0.059 (0.018) | 0.055 (0.018) | 0.053 (0.018) | 0.036 (0.018) |
|  | t = 4.387 | t = 3.337 | t = 3.109 | t = 2.997 | t = 1.982 |
|  | p < 0.001 | p = 0.001 | p = 0.002 | p = 0.003 | p = 0.048 |
| **Male sex** | 0.273 (0.016) | 0.270 (0.017) | 0.266 (0.017) | 0.267 (0.017) | 0.262 (0.017) |
|  | t = 16.966 | t = 16.210 | t = 16.974 | t = 16.129 | t = 15.684 |
|  | p < 0.001 | p < 0.001 | p < 0.001 | p < 0.001 | p < 0.001 |
| **Asian** |  | -0.060 (0.058) | -0.034 (0.058) | -0.032 (0.058) | -0.035 (0.060) |
|  |  | t = -1.033 | t = -0.579 | t = -0.548 | t = -0.572 |
|  |  | p = 0.302 | p = 0.563 | p = 0.584 | p = 0.568 |
| **Black** |  | -0.119 (0.059) | -0.082 (0.058) | -0.081 (0.058) | -0.072 (0.060) |
|  |  | t = -2.034 | t = -1.400 | t = -1.396 | t = -1.205 |
|  |  | p = 0.043 | p = 0.162 | p = 0.163 | p = 0.229 |
| **Non-Irish White** |  | 0.039 (0.028) | 0.051 (0.027) | 0.056 (0.028) | 0.061 (0.028) |
|  |  | t = 1.421 | t = 1.847 | t = 2.029 | t = 2.176 |
|  |  | p = 0.156 | p = 0.065 | p = 0.043 | p = 0.03 |
| **Income** |  | -0.009 (0.003) | -0.007 (0.003) | -0.007 (0.003) | -0.007 (0.004) |
|  |  | t = -2.644 | t = -1.994 | t = -1.949 | t = -1.990 |
|  |  | p = 0.009 | p = 0.047 | p = 0.052 | p = 0.047 |
| **Education** |  | -0.040 (0.008) | -0.037 (0.008) | -0.036 (0.008) | -0.033 (0.008) |
|  |  | t = -4.992 | t = -4.670 | t = -4.506 | t = -3.998 |
|  |  | p < 0.001 | p < 0.001 | p < 0.001 | p < 0.001 |
| **Single-parent** |  | -0.113 (0.027) | -0.092 (0.027) | -0.091 (0.027) | -0.073 (0.028) |
|  |  | t = -4.208 | t = -3.375 | t = -3.363 | t = -2.650 |
|  |  | p < 0.001 | p = 0.001 | p = 0.001 | p = 0.009 |
| **Maternal age** |  | -0.014 (0.002) | -0.013 (0.002) | -0.013 (0.002) | -0.013 (0.002) |
|  |  | t = -8.221 | t = -8.071 | t = -7.896 | t = -7.536 |
|  |  | p < 0.001 | p < 0.001 | p < 0.001 | p < 0.001 |
| **Parental depression/anxiety** |  |  | 0.143 (0.018) | 0.138 (0.018) | 0.132 (0.018) |
|  |  |  | t = 8.166 | t = 7.873 | t = 7.446 |
|  |  |  | p < 0.001 | p < 0.001 | p < 0.001 |
| **Parental conduct problems** |  |  | 0.035 (0.023) | 0.034 (0.023) | 0.029 (0.023) |
|  |  |  | t = 1.504 | t = 1.478 | t = 1.237 |
|  |  |  | p = 0.133 | p = 0.140 | p = 0.217 |
| **Familial substance-use** |  |  | 0.174 (0.061) | 0.162 (0.06) | 0.132 (0.063) |
|  |  |  | t = 2.856 | t = 2.644 | t = 2.110 |
|  |  |  | p = 0.005 | p = 0.009 | p = 0.035 |
| **Total pregnancy Complications** |  |  |  | 0.029 (0.009) | 0.033 (0.010) |
|  |  |  |  | t = 3.044 | t = 3.437 |
|  |  |  |  | p = 0.003 | p = 0.001 |
| **Smoking in pregnancy** |  |  |  |  | 0.099 (0.025) |
|  |  |  |  |  | t = 3.988 |
|  |  |  |  |  | p < 0.001 |
| **Alcohol in pregnancy** |  |  |  |  | 0.045 (0.020) |
|  |  |  |  |  | t = 2.194 |
|  |  |  |  |  | p = 0.029 |
| **Drugs in pregnancy** |  |  |  |  | 0.071 (0.084) |
|  |  |  |  |  | t = 0.840 |
|  |  |  |  |  | p = 0.402 |
| **Observations** | 7627 | 6920 | 6918 | 6916 | 6731 |
| **R^2^** | 0.037 | 0.067 | 0.078 | 0.079 | 0.083 |
| **Adjusted R^2^** | 0.037 | 0.066 | 0.077 | 0.078 | 0.081 |

**Table S6.** Results of ordinal logistic modelling in GUI (Ireland)

|  | ***Dependent variable: ADHD symptom group (low, moderate, high, very high)*** | | | | |
| --- | --- | --- | --- | --- | --- |
|  | **baseline** | **+SES+Demo** | **+famhx** | **+complications** | **+substances** |
| **Restricted Fetal Growth** | 0.185 (0.046) | 0.147 (0.049) | 0.135 (0.050) | 0.129 (0.050) | 0.091 (0.051) |
|  | t = 4.010 | t = 2.978 | t = 2.729 | t = 2.609 | t = 1.789 |
|  | p < 0.001 | p = 0.003 | p = 0.007 | p = 0.01 | p = 0.074 |
| **Male sex** | 0.740 (0.044) | 0.753 (0.047) | 0.755 (0.047) | 0.758 (0.047) | 0.749 (0.047) |
|  | t = 16.828 | t = 16.195 | t = 16.176 | t = 16.240 | t = 15.798 |
|  | p < 0.001 | p < 0.001 | p < 0.001 | p < 0.001 | p < 0.001 |
| **Asian** |  | -0.307 (0.161) | -0.232 (0.163) | -0.227 (0.163) | -0.190 (0.170) |
|  |  | t = -1.909 | t = -1.424 | t = -1.397 | t = -1.114 |
|  |  | p = 0.057 | p = 0.155 | p = 0.163 | p = 0.266 |
| **Black** |  | -0.186 (0.159) | -0.185 (0.159) | -0.082 (0.159) | -0.069 (0.164) |
|  |  | t = -1.169 | t = -0.535 | t = -0.515 | t = -0.422 |
|  |  | p = 0.243 | p = 0.593 | p = 0.607 | p = 0.674 |
| **Non-Irish White** |  | 0.072 (0.075) | 0.106 (0.075) | 0.120 (0.076) | 0.125 (0.078) |
|  |  | t = 0.956 | t = 1.401 | t = 1.591 | t = 1.609 |
|  |  | p = 0.339 | p = 0.162 | p = 0.112 | p = 0.108 |
| **Income** |  | -0.02 (0.010) | -0.014 (0.010) | -0.014 (0.010) | -0.014 (0.010) |
|  |  | t = -2.117 | t = -1.476 | t = -1.440 | t = -1.452 |
|  |  | p = 0.035 | p = 0.141 | p = 0.150 | p = 0.147 |
| **Education** |  | -0.108 (0.022) | -0.101 (0.022) | -0.097 (0.022) | -0.092 (0.023) |
|  |  | t = -4.855 | t = -4.534 | t = -4.348 | t = -4.013 |
|  |  | p < 0.001 | p < 0.001 | p < 0.001 | p < 0.001 |
| **Single-parent** |  | -0.317 (0.073) | -0.254 (0.075) | -0.251 (0.074) | -0.208 (0.076) |
|  |  | t = -4.320 | t = -3.413 | t = -3.380 | t = -2.745 |
|  |  | p < 0.001 | p = 0.001 | p = 0.001 | p = 0.007 |
| **Maternal age** |  | -0.033 (0.005) | -0.032 (0.005) | -0.032 (0.005) | -0.032 (0.005) |
|  |  | t = -7.191 | t = -7.064 | t = -6.914 | t = -6.639 |
|  |  | p < 0.001 | p < 0.001 | p < 0.001 | p < 0.001 |
| **Parental depression/anxiety** |  |  | 0.380 (0.049) | 0.367 (0.049) | 0.362 (0.050) |
|  |  |  | t = 7.825 | t = 7.537 | t = 7.305 |
|  |  |  | p < 0.001 | p < 0.001 | p < 0.001 |
| **Parental conduct problems** |  |  | 0.087 (0.063) | 0.087 (0.063) | 0.077 (0.065) |
|  |  |  | t = 1.374 | t = 1.370 | t = 1.198 |
|  |  |  | p = 0.170 | p = 0.171 | p = 0.231 |
| **Familial substance-use** |  |  | 0.556 (0.167) | 0.533 (0.169) | 0.456 (0.175) |
|  |  |  | t = 3.325 | t = 3.163 | t = 2.607 |
|  |  |  | p = 0.001 | p = 0.002 | p = 0.010 |
| **Total pregnancy Complications** |  |  |  | 0.079 (0.026) | 0.088 (0.027) |
|  |  |  |  | t = 2.990 | t = 3.277 |
|  |  |  |  | p = 0.003 | p = 0.002 |
| **Smoking in pregnancy** |  |  |  |  | 0.233 (0.069) |
|  |  |  |  |  | t = 3.398 |
|  |  |  |  |  | p = 0.001 |
| **Alcohol in pregnancy** |  |  |  |  | 0.104 (0.057) |
|  |  |  |  |  | t = 1.817 |
|  |  |  |  |  | p = 0.07 |
| **Drugs in pregnancy** |  |  |  |  | 0.114 (0.221) |
|  |  |  |  |  | t = 0.518 |
|  |  |  |  |  | p = 0.605 |
| **Observations** | 7627 | 6920 | 6918 | 6916 | 6731 |

## Further details on familial factors

### Household Income

ABCD, the primary respondent was asked: “*What is your total combined family income for the past 12 months? This should include income (before taxes and deductions) from all sources, wages, rent from properties, social security, disability and/or veteran's benefits, unemployment benefits, workman's compensation, help from relative (include child payments and alimony), and so on.*”. Ten income brackets were provided as outcomes: <$5,000; $5,000-11,999; $12,000-15,999; $16,000-24,999; $25,000-34,999; $35,000-49,999; $50,000-74,999; $75,000-99,999; $100,000-199,999; $200,000+. Household income in ABCD therefore refers to the household income *bracket* reflecting income from all sources (and *before* deductions).

In GUI, the primary respondant was asked “*If you added up all the income sources from ALL household members what would be the total household net income, i.e. after deductions for tax and PRSI* [social insurance] *only? Include income from all sources and from all household members*”. This figure was subsequently equivalized i.e. divided by the number of people living in the household and made into deciles. An equivalence scale was used to assign a “weight” to each household member (weight of 1 to the first adult in the household, 0.66 to each subsequent adult aged 14+ and 0.33 to each child <14 years). Household income in GUI therefore refers to the household *equivalised* income from all sources (and *after* deductions) in *deciles*.

Household income was treated as a continuous fixed effect in the analysis.

### Parental Education

The dominance criterion was applied to education status of both parents, such that the highest education level achieved (of either parent) becomes the education status of the household. If there was missing data on the highest education level attained for one parent, the household education status became the education level of the other parent. This variable was treated as a continuous fixed effect in the analysis, in line with the assumption that parental educational level would be linearly related to the quality of the child’s mental health.

The 21 levels of education originally in ABCD (US) were simplified into 5 levels: (1) Incomplete Schooling (grades 1-12 without graduation); (2) High School Degree/GED (includes partial college); (3) Associate Degree (occupational/academic); (4) Bachelor or Professional Degree; (5) Postgraduate Degree (e.g. Masters, Doctorate).

The 13 levels of education originally recorded in GUI (Ireland) were simplified into 5 levels: (1) Incomplete Schooling (no formal education/primary only/lower secondary e.g. Junior Certificate); (2) Secondary School Degree (e.g.upper secondary graduation e.g. Leaving Certificate/technical or vocational qualification/both); (3) Non-degree Qualification (4) Primary/Professional Degree (or both); (5) Postgraduate Cert, Diploma or Degree.

### Single-parenthood

In ABCD, single-parenthood was established by the question “do you have a partner?” to the primary respondent (most often the mother) and partner was defined as anyone who helps in raising the child or has helped for more than 2 years. This person had to be involved 40% or more of the child’s daily activities and could be a spouse, boyfriend/girlfriend, relative or friend of the parent.

In GUI, single-parenthood was established by the question “Do you have a resident spouse / partner” to the primary caregiver (most often the mother). This variable was cross-validated with another variable created by the data processors indicating whether the household was a one-parent or two-parent household (hhtype4).

### Family Psychiatric History

In ABCD, primary respondent was asked whether either biological parent had ever experienced (1) depression “suffered from depression, that is, have they felt so low for a period of at least two weeks that they hardly ate or slept or couldn't work or do whatever they usually do?” or (2) problems with nerves “had any other problems with their nerves, or had a nervous breakdown?”. “Parental depression/anxiety” was endorsed if either parent reported either condition. Similarly, “parental conduct issues” was endorsed if either parent was reported to match the following description “the kind of person who never holds a job for long, or gets into fights, or gets into trouble with the police from time to time, or had any trouble with the law as a child or an adult?”.

In GUI, both parents were asked whether they had been treated by a medical professional for clinical depression, anxiety or “nerves”. They were asked a maximum of 4 times across the various waves of data collection (at child age: 9 months, 3, 5 and 9 years). If *either* parent said yes at *any* of these waves, the “parental depression/anxiety” variable was endorsed for this household. The same aggregation of data across these waves was performed for a more accurate reading of lifetime parental conduct issues “trouble with the [police] other than for traffic offences”. Data on substance-use issues in the family was available at two waves (child ages 5 and 9). Endorsement of this item at either timepoint resulted in an endorsement of this variable.

**Table S7.** Cohort-specific definitions for each aspect of family psychiatric history (parent-reported)

|  | **ABCD Definition** | **% Yes (n)** | **GUI Definition** | **% Yes (n)** |
| --- | --- | --- | --- | --- |
| **Parental depression/anxiety** | At least one parent has suffered from depression, had problems with nerves or had a nervous breakdown (lifetime) | 34% (3295) | At least one parent has been treated for clinical depression, anxiety, nerves of phobias (lifetime) | 34% (2694) |
| **Parental conduct issues** | At least one parent is the type of person who doesn’t hold a job for long, gets into fights, has had trouble with the police or with the law (lifetime) | 14% (1314) | At least one parent has been in trouble with the Gardaí (Irish police) or police in another country, other than for traffic offences (lifetime) | 15% (1192) |
| **Familial substance-use issues** | At least one member of the immediate family (biological parents or full-siblings) has a history of drug/alcohol-related problems (lifetime) | 20% (1966) | Study child has experienced alcoholism or drug-use within the immediate family (*child’s* lifetime) | 2% (161) |

## Maternal smoking in pregnancy

**Table S8.** Timing and frequency of maternal smoking during pregnancy for both cohorts

| **ABCD** | | | |
| --- | --- | --- | --- |
|  | **Before knowing of pregnancy** | **After knowing of pregnancy** |  |
| **% smoked (n)** | 12.3% (1087) | 4.3% (383) |  |
| **Cigarettes per day** |  |  |  |
| Min-Max | 0-50 | 1-30 |  |
| Mean | 8.24 | 7.53 |  |
| Standard Deviation | 6.27 | 5.70 |  |
| **GUI** | | | |
|  | **Trimester 1** | **Trimester 2** | **Trimester 3** |
| **% smoked (n)** | 13.5% (1045) | 12.3% (951) | 12.1% (938) |
| **Cigarettes per day** |  |  |  |
| Min-Max | 1-56 | 1-45 | 1-60 |
| Mean | 9.09 | 9.11 | 8.99 |
| Standard Deviation | 6.51 | 6.44 | 6.64 |

**Figure S5.** Distributions of fetal growth in the ABCD and GUI samples. Fetal growth is measured in kilograms (KGs) from the average birth weight for one’s gestational age. Also indicated are SGA and LGA groups defined as 2 standard deviations below and above the mean birth weight for one’s gestational age respectively.


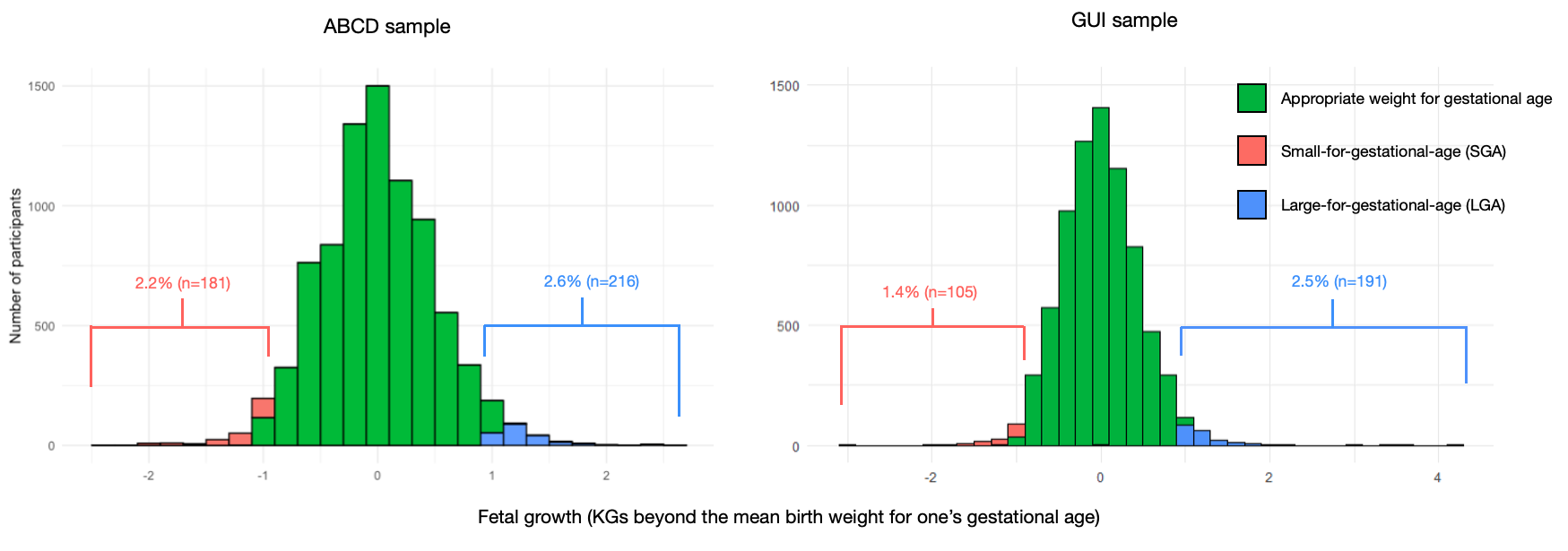


**Figure S6.** Fetal growth in ABCD and GUI samples compared to World Health Organisation (WHO) norms. WHO fetal growth curve refers to the median of 10 high-middle income countries (singleton pregnancies only), extracted from data in Kiserud et al. (2017)*.


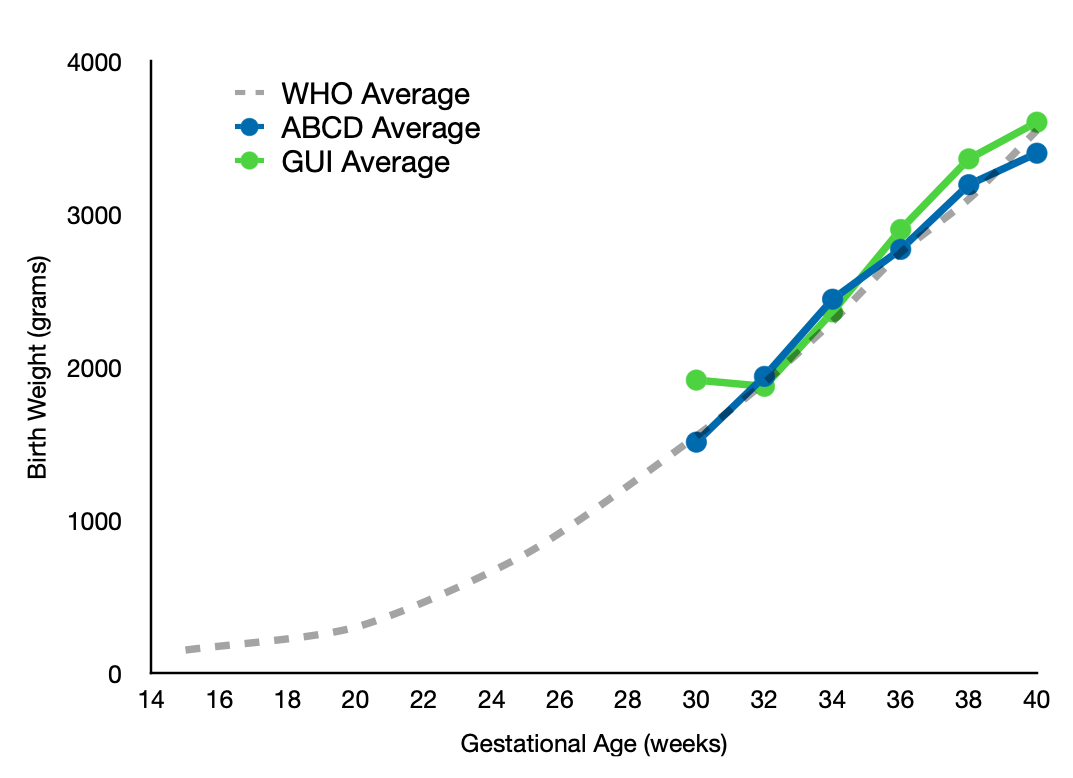


*Kiserud, T., Piaggio, G., Carroli, G., Widmer, M., Carvalho, J., Neerup Jensen, L., Giordano, D., Cecatti, J. G., Abdel Aleem, H., & Talegawkar, S. A. (2017). The World Health Organization fetal growth charts: a multinational longitudinal study of ultrasound biometric measurements and estimated fetal weight. *PLoS medicine, 14*(1), e1002220.
